# Carcinoma of the larynx, metastatic to illeum, presents as ruptured appendicitis: case report and literature review

**DOI:** 10.1186/1916-0216-43-18

**Published:** 2014-06-25

**Authors:** Jordan T Glicksman, David Bottoni, Jessica Shepherd, Neil Parry, Jason H Franklin

**Affiliations:** 1 Department of Otolaryngology—Head and Neck Surgery, Schulich School of Medicine, University of Western Ontario, London, Ontario, Canada; 2 Division of General Surgery and Division of Critical Care, Schulich School of Medicine, University of Western Ontario, London, Ontario, Canada; 3 Department of Pathology, Schulich School of Medicine, University of Western Ontario, London, Ontario, Canada; 4 Department of Otolaryngology, Queens University, Kinston, Ontario, Canada; 5 Hotel Dieu Hospital, Murray Building, Kingston, Ontario, Canada

**Keywords:** Larynx, Squamous cell carcinoma, Metastasis, Small intestine, Perforation

## Abstract

**Objectives:**

Metastasis of laryngeal squamous cell carcinoma (SCC) to the intra-abdominal gastrointestinal tract is exceedingly rare. The objectives of this case report are to describe a case involving a perforated metastasis of a laryngeal SCC to the ileum and to review the literature pertaining to other similar cases.

**Methods:**

A review of patient’s chart and a review of the English literature involving malignant SCC of the larynx with metastasis to the small bowel.

**Results:**

We describe the case of a 58-year-old man who had failed induction chemotherapy and underwent a laryngopharyngectomy with bilateral neck dissection and pectoralis major flap for a T4N2c laryngeal SCC. Subsequently, the patient was treated with postoperative radiation and cituximab.

The patient went on to present with symptoms consistent with a ruptured appendix, supported by ultrasound imaging. The patient was taken to the operating room where a right hemicolectomy was performed. Pathological gross examination confirmed a 4 cm transmural perforation in the terminal ileum. Microscopy demonstrated deposits of metastatic squamous cell carcinoma in the surrounding smooth muscle. Metastatic carcinoma was also found in a separate nodule from the abdominal wall. The patient had an uncomplicated post-operative period, and survived several months thereafter.

**Conclusions:**

Metastasis of laryngeal SCC to the small bowel with perforation is exceedingly rare, but possible. These patients may be successfully managed with resection.

## Background

Squamous Cell Carcinoma (SCC) of the head and neck represents the sixth most common cancer in the world [1,2]. Among head and neck SCC, the larynx is second to the oral cavity as the most common site of malignancy [3,4]. The typical pattern of spread for laryngeal SCC includes metastasis to local and mediastinal lymph nodes, lungs, liver and bone [5]. While metastatic tumors derived from other organs such as the uterus, ovaries, lung and testes as well as melanomas frequently metastasize to the small bowel, reports of laryngeal cancer metastasizing to this site are exceedingly rare. We describe the case of a patient with a small bowel perforation caused by a metastatic deposit of laryngeal SCC.

## Case presentation

A 58 year-old male presented to a community hospital with an acute onset of right lower quadrant pain and tenderness over McBurney’s point. The patient had a known history of T4N2c squamous cell carcinoma of the larynx. The patient had previously failed induction chemotherapy and underwent a laryngopharyngectomy with bilateral neck dissection and pectoralis major flap five and a half months prior to presentation, which was followed by postoperative radiation and chemotherapy.

An ultrasound performed at the hospital of presentation confirmed suspicion of acute appendicitis. Laboratory investigations revealed an elevated white blood cell count, a normal lactate and were otherwise unremarkable. The patient was accepted for transfer to our institution for a planned laparoscopic appendectomy for which he was taken to the operating room upon confirmation of his clinical findings.

**Figure 1:**
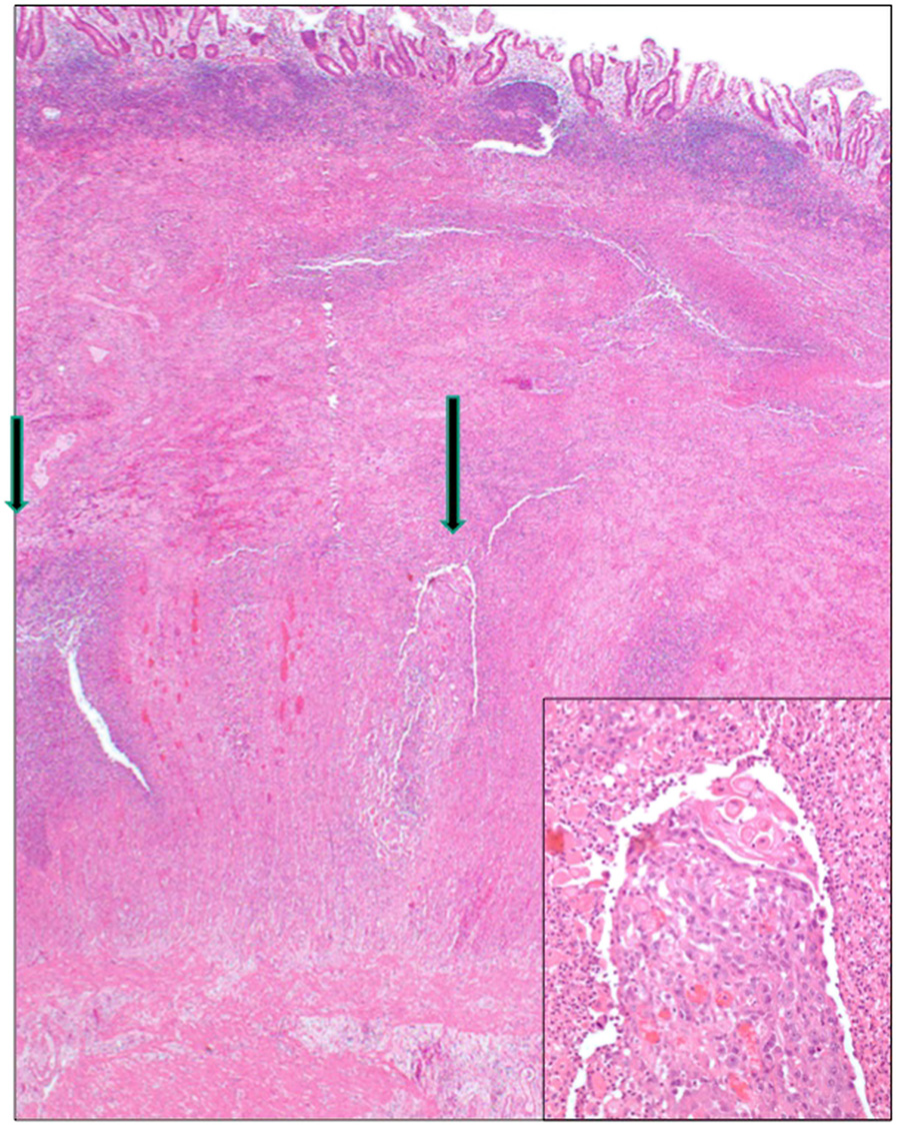
**Metastatic squamous cell carcinoma in ileal muscle coat (arrows).** Inset: high power view of right arrow region.

**Figure 2:**
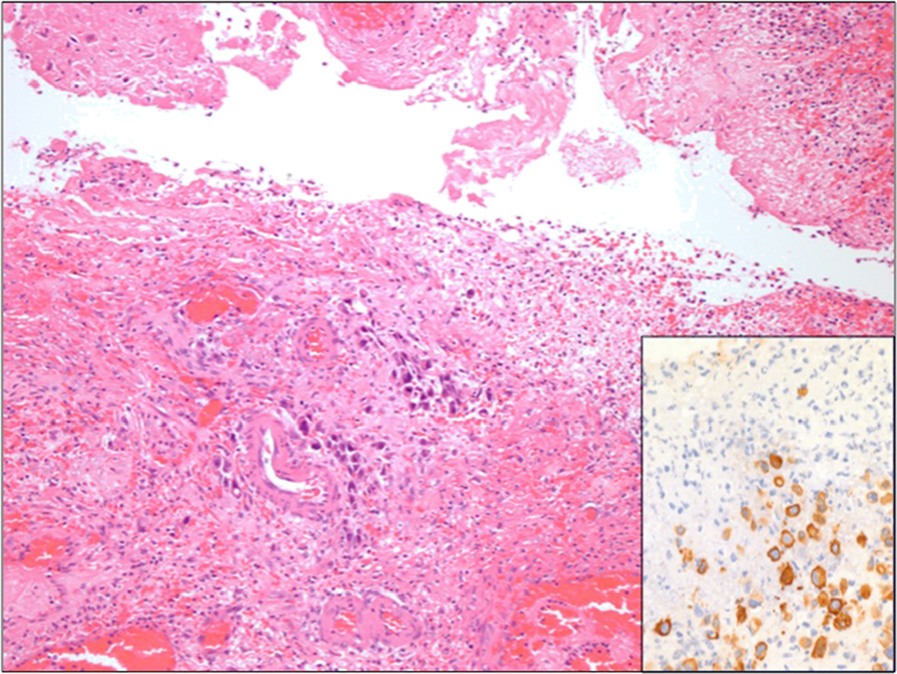
**Metastatic tumor in ileal perforation site.** Inset: Cytokeratin (CK34BE12) immunohistochemical stain.

Upon laparoscopy, visualization of the appendix was limited by inflammation of the cecum and ileocecal junction and so conversion to an open procedure occurred through a midline incision. A perforated tumor of the distal ileum was quickly revealed for which a formal right hemicolectomy was performed. The abdomen was irrigated with normal saline before closure.Pathological gross examination confirmed a 4 cm transmural perforation in the terminal ileum, with an ill-defined nodular area at its distal edge. There was also an area of thicker serosal exudate around the tip of the appendix. Microscopy demonstrated deposits of metastatic squamous cell carcinoma in the muscle wall of the terminal ileum (Figure 1) and the mesoappendix. Degenerating tumor cells were within the ileal perforation tract (Figure 2). There was no appendicitis or appendiceal perforation. Metastatic carcinoma was also found in a separate nodule from the abdominal wall.

The patient survived the procedure and post-operative period which was uncomplicated. He received a targeted EGFR inhibitor and was symptom free. Unfortunately, the patient was readmitted to hospital within five months with widespread metastases and pneumonia. He died shortly thereafter secondary to respiratory failure.

## Discussion

Primary tumors that commonly metastasize to the small intestine include those of uterine, ovarian, lung and testicular origin as well as melanoma. Metastasis to the small intestine can occur by contiguous spread, peritoneal metastases or hematogenous metastases and can be difficult to detect until they progress sufficiently to cause symptoms such as obstruction, bleeding or perforation [6].

Laryngeal SCC seldom metastasizes to the small bowel. A recent review of all published cases of SCC from head and neck cancer sites by Dwivedi et al. revealed only 12 such cases, of which only nine cases were derived from a laryngeal primary neoplasm [7]. In their review they describe three cases in which a laryngeal SCC metastasis caused intestinal obstruction, two cases of bleeding or melena, one of biliary obstruction and three cases of small bowel perforation [8-17].

## Conclusion

To the best of our knowledge our case represents the tenth documented case of laryngeal cancer metastasizing to the small intestine and only the fourth case of a laryngeal SCC metastasis resulting in small bowel perforation. Our case is interesting in that proximity of the deposit to the appendix resulted in both a clinical and radiologic presentation consistent with that of acute ruptured appendicitis, which was certainly more likely, even in the context of the patient’s prior cancer history. Our case is unique as the patient underwent resection and remained well and symptom free for 5 months after potentially life-threatening metastasis and visceral perforation. This case affirms that in the context of a patient presenting with symptoms consistent with bowel perforation with a past history of advanced laryngeal cancer, perforation secondary to tumor metastasis may be considered.

“This material has never been published and is not currently under evaluation in any other peer-reviewed publication”.

## Consent

Written informed consent was obtained from the patient for the publication of this report and any accompanying images.

## Competing interests

The authors declare that they have no competing interests.

## Authors’ contribution

All authors contributed equally to this manuscript. All authors have read and approved the final manuscript.
